# Spatial Landscape of Malignant Pleural and Peritoneal Mesothelioma Tumor Immune Microenvironments

**DOI:** 10.1158/2767-9764.CRC-23-0524

**Published:** 2024-08-16

**Authors:** Xiaojun Ma, David Lembersky, Elena S. Kim, Michael J. Becich, Joseph R. Testa, Tullia C. Bruno, Hatice U. Osmanbeyoglu

**Affiliations:** 1 UPMC Hillman Cancer Center, Cancer Biology Program, University of Pittsburgh School of Medicine, Pittsburgh, Pennsylvania.; 2 Department of Biomedical Informatics, University of Pittsburgh School of Medicine, Pittsburgh, Pennsylvania.; 3 Cancer Prevention and Control Program, Fox Chase Cancer Center, Philadelphia, Pennsylvania.; 4 Department of Immunology, University of Pittsburgh School of Medicine, Pittsburgh, Pennsylvania.; 5 Department of Bioengineering, University of Pittsburgh School of Engineering, Pittsburgh, Pennsylvania.; 6 Department of Biostatistics, University of Pittsburgh School of Public Health, Pittsburgh, Pennsylvania.

## Abstract

Immunotherapies have demonstrated limited clinical efficacy in malignant mesothelioma treatment. We conducted multiplex immunofluorescence analyses on tissue microarrays (*n* = 3) from patients with malignant pleural mesothelioma (MPM, *n* = 88) and malignant peritoneal mesothelioma (MPeM, *n* = 25). Our study aimed to elucidate spatial distributions of key immune cell populations and their association with lymphocyte activation gene 3 (LAG3), BRCA1-associated protein 1 (BAP1), neurofibromatosis type 2 (NF2), and methylthioadenosine phosphorylase (MTAP), with MTAP serving as a cyclin-dependent kinase inhibitor 2A/2B (CDKN2A/B) surrogate marker. Additionally, we examined the relationship between the spatial distribution of major immune cell types and prognosis and clinical characteristics of patients with malignant mesothelioma. We observed a higher degree of interaction between immune cells and tumor cells in MPM compared with MPeM. Notably, within MPM tumors, we detected a significantly increased interaction between tumor cells and CD8^+^ T cells in tumors with low BAP1 expression compared with those with high BAP1 expression. To support the broader research community, we have developed The Human Spatial Atlas of Malignant Mesothelioma, containing hematoxylin and eosin and multiplex immunofluorescence images with corresponding metadata.

**Significance:**

Considering the limited therapeutic options available to patients with malignant mesothelioma, there is substantial translational potential in understanding the correlation between the spatial architecture of the malignant mesothelioma tumor immune microenvironment and tumor biology. Our investigation reveals critical cell–cell interactions that may influence the immune response against malignant mesothelioma tumors, potentially contributing to the differential behaviors observed in MPM and MPeM. These findings represent a valuable resource for the malignant mesothelioma cancer research community.

## Introduction

Malignant mesothelioma, a rare and highly aggressive cancer originating from serosal surfaces and strongly associated with asbestos exposure (1, 2), poses significant challenges in both clinical management and understanding. Malignant mesothelioma primarily manifests in the pleura (lining of the lungs) but can manifest in various other anatomic locations, including the peritoneum (abdominal lining), pericardium (heart lining), and tunica vaginalis (covering of the testicles). Malignant pleural mesothelioma (MPM) accounts for approximately 80% of malignant mesothelioma cases, whereas malignant peritoneal mesothelioma (MPeM) constitutes approximately 15% to 20% (3). Histologically, malignant mesothelioma can be categorized into three primary subtypes, each exhibiting distinct biological and clinical characteristics: epithelioid (the most common type), sarcomatoid, and biphasic (a combination of epithelioid and sarcomatoid features).

A striking disparity in prognosis exists between the two major malignant mesothelioma subtypes. MPeM presents a significantly more favorable prognosis than MPM. In cases amenable to cytoreductive surgery and hyperthermic intraperitoneal chemotherapy, patients with MPeM achieve a median overall survival (OS) exceeding 5 years (3). Conversely, MPM continues to pose a clinical challenge, with a dismal 5-year survival rate of less than 10% (4). The historical gender-based risk difference, with men being at higher risk due to occupational asbestos exposure, is shifting. Malignant mesothelioma diagnoses now include younger individuals and women with no known history of asbestos exposure (5, 6). This evolving epidemiologic pattern underscores the need for comprehensive research to better understand the disease’s complexities and improve treatment strategies.

Malignant mesothelioma remains an incurable malignancy. Currently, approved first-line therapies for MPM include immunotherapy (nivolumab plus ipilimumab) and chemotherapy (cisplatin plus pemetrexed), but their impact on OS is modest, offering only month-long extensions of life expectancy (7, 8). This limited therapeutic success may be attributed, in part, to the complex structure of the malignant mesothelioma tumor immune microenvironment (TIME). Recent investigations into potential biomarkers have shed light on this challenge. Notably, exploratory analyses have revealed that patients with MPM treated with immunotherapy may experience prolonged median OS when exhibiting an inflammatory gene signature score. The signature score, based on the levels of key biomarkers such as CD8A, CD274/PDL1, STAT1, and lymphocyte activation gene 3 (LAG3), seems to influence OS independently of histologic subtype (9).

In recent decades, progress has been made in unraveling the molecular mechanisms underlying the development and progression of malignant mesothelioma. MPM and MPeM share common cellular features and genetic alterations. The genomic profile of malignant mesothelioma highlights a relatively low mutation rate in protein-coding regions, with the most prevalent alterations being mutations and deletions within critical tumor suppressor genes (TSGs). Among these, BRCA1-associated protein 1 (*BAP1*) located on chromosome band 3p21, neurofibromatosis type 2 (*NF2*) on 22q12, and cyclin-dependent kinase inhibitor 2A/2B (*CDKN2A/B*) on 9p21 are frequently affected (10–15).

Our comprehensive analysis focused on MPM tumors harboring genomic alterations within one or more of these TSGs, which are considered critical drivers in MPM pathogenesis (16). Although alterations of these key TSGs frequently occur in various combinations in a given malignant mesothelioma tumor, we found that alterations in *BAP1* alone were strongly associated with improved clinical outcomes and the emergence of a distinctive immunotherapy response signature. Recent research has revealed that *BAP1* alterations correlate with perturbed immune signaling in MPeM (17). Additionally, MPM tumors carrying alterations in *BAP1* alone exhibit a distinctive gene expression pattern in the context of the inflammatory TIME. This pattern includes the activation of IFN signaling and heightened expression of immune checkpoints such as *LAG3* and V-domain Ig suppressor of T-cell activation (*VISTA*; ref. 16). *LAG3*, an immune checkpoint primarily expressed on activated T cells, has now become a vital component of combinatorial immunotherapies utilized in the treatment of metastatic melanoma (18).

A comprehensive understanding of the TIME and its associated molecular and clinical characteristics, differentiating the two major malignant mesothelioma subtypes, is crucial for the development of effective treatment. Spatial technologies have recently provided unprecedented insights into the intricate complexities of the TIME with significant prognostic and predictive values. The primary objective of this study was to advance our understanding of the malignant mesothelioma TIME while examining the protein expression levels for LAG3, BAP1, NF2, and methylthioadenosine phosphorylase (MTAP). MTAP served as a surrogate marker for CDKN2A/B, as the *MTAP* gene, located at 9p21, is frequently codeleted with *CDKN2A/B* within distinct types of malignant mesothelioma. To achieve this, we used multiplex immunofluorescence (mIF) techniques applied to tissue microarray (TMA) sections from both MPM and MPeM samples.

## Materials and Methods

### National Mesothelioma Virtual Bank TMAs

We obtained malignant mesothelioma TMAs consisting of three sets totaling 336 sample cores, predominantly comprising epithelioid histology from 115 patients from the National Mesothelioma Virtual Bank (NMVB; Supplementary Table S1). These TMAs were prepared by the Roswell Park Cancer Institute, University of Pennsylvania, and University of Pittsburgh. Consolidated deidentified patient data were also provided by the NMVB. Briefly, the cases were reviewed by a pathologist at each collaborative institute, and selected tumors were marked to identify areas with tumor while preparing NMVB TMAs. Marking of areas with a significant fraction of tumor cells was performed by board-certified surgical pathologists, and the markings were checked before TMA production was initiated. If there were benign areas within the cancer, these areas were marked out so no punches were taken from those areas. If the cancerous area was small, a small arrow was placed to direct the punch. The NMVB prepared the TMAs from formalin-fixed, paraffin-embedded malignant mesothelioma tissue blocks that were sectioned, placed on positively charged slides, and then stored in green polyethylene microcline plastic boxes (Thermo Fisher Scientific, Cat. # 03-448-5) in a vacuum desiccator at 4°C in the dark. TMA production was accomplished using a Beecher TMA with associated simple automated changes (19). Sectioning was performed using a standard microtome with a collimator to improve lock alignment and minimize tissue loss. When a request for TMA slides was approved by the NMVB, the NMVB’s TMA coordinator arranged the expedited temperature-controlled transportation for further histologic analysis.

### IHC

Hematoxylin and Eosin (H&E) staining on TMAs was performed at the Research Developmental Lab, Department of Pathology, University of Pittsburgh. TMAs were stained using the following protocol. Initially, the slides were baked at 60°C for 1 hour. The slides were then deparaffinized using xylene (Fisher Chemical, #X3P-1GAL) in three changes for 5 minutes each. This was followed by rehydration in 100% ethanol for 5 minutes (two changes), 95% ethanol for 5 minutes (two changes), and distilled water (dH_2_0) with 10 to 15 dips (two changes). Hematoxylin staining was achieved using SelecTech Hematoxylin 560 MX (Leica Biosystems, #3801575) for 8 minutes. The slides were then rinsed in dH_2_O five times. Bluing was carried out using SelecTech Define MX-aq (Leica Biosystems, #3803598) for 10 seconds, followed by 10 dips in dH_2_O, a 20-second treatment with SelecTech Blue Buffer 8 (Leica Biosystems, #3802918), and another 10 dips in dH_2_O. For eosin staining, the slides were dipped in 95% ethanol 10 times before being stained with SelecTech Eosin Phloxine 515 (Leica Biosystems, #3801606) for 20 seconds, followed by dehydration via five dips in 95% ethanol and 10 dips in 100% ethanol (two changes). The slides were then cleared in xylene (three changes, 10–15 dips each) before being cover-slipped. Digital images of these slides were scanned at 400× magnification on Aperio AT2 (Leica).

### Multiplex immunofluorescence (mIF)

We conducted mIF on the NMVB TMAs. Multiplexed immunostaining of the TMAs was performed using the manufacturer’s (Akoya Biosciences) protocol with the following antibodies: FOXP3: 1:200 (Cell Signaling Technology, Cat# 98377S, Clone D2W8E), CD8: 1:150 (Biocare Medical, Cat# ACI 3160A, Clone C8/144B), CD68: 1:800 (Cell Signaling Technology, Cat# 76437S, Clone D4B96), pan-cytokeratin (CK): 1:150 (Santa Cruz, Cat# sc-81714, Clone AE1/AE3), CD20: 1:300 (Leica Biosystems, Cat# NCL-L-CD20-L26, Clone L26), and CD4: prediluted (Biocare Medical, Cat# API3209AA, Clone EP204), LAG3: 1:250 (Cell Signaling Technology, Cat# 45208S, Clone D2G4O), CD11c: 1:400 (Cell Signaling Technology, Cat# 45581S, Clone D3V1E), CD56: 1:100 (Biocare Medical, Cat# CM164A, Clone BC56C04), BAP1: 1:100 (Biocare Medical, Cat# ACI3247A), MTAP: 1:500 (Abcam, Cat# Ab96231), and NF2: 1:300 (Sigma, Cat# HPA003097). Cell nuclei were detected with DAPI (AkoyaBio kit).

Two rounds/panels of multiplex immunofluorescence (mIF) staining were conducted on sets of corresponding TMAs. In the first panel, we used antibodies targeting pan-CK (epithelial cell marker), CD4 (helper T-cell marker), CD8 (cytotoxic T-cell marker), CD20 (B-cell marker), CD68 (macrophage marker), and FOXP3 (regulatory T-cell (Treg) marker). Subsequently, a second round of mIF staining was performed with antibodies against CD11c [dendritic cell (DC) marker], CD56 (NK cell marker), BAP1, MTAP, LAG3, and NF2.

Individual antibodies were first tested as single stains according to the suppliers’ recommendations using appropriate control tissues before beginning the multiplex panel optimization. For multiplex staining, Akoya Bioscience’s Opal 6-Plex detection kit was used according to the manufacturer’s instructions (Cat# NEL871001KT). The immunophenotyping panel was adapted from Onkar and colleagues (20). The panel was reoptimized for Akoya’s MOTiF platform, which minimizes spectral overlap of fluorophores. Optimization and validation steps were performed in collaboration with the University of Pittsburgh Medical Center Hillman Cancer Center Translational Pathology Imaging Laboratory using healthy donor lymphoid tissue as a control and for validation (Supplementary Fig. S1). Automated staining of tissues was performed using the Leica Bond RX. Briefly, tissue slides were processed as follows: baked in a dry oven at 60°C, deparaffinized with xylene and ethanol, and refixed in 10% neutral-buffered formalin for 15 minutes. Heat-induced antigen retrieval was performed using microwave heating, followed by blocking for 10 minutes. Slides were incubated with primary antibodies for 30 minutes at room temperature. Secondary antibodies conjugated to horseradish peroxidase were added for 10 minutes. Cells were stained with the following primary antibody/conjugated Opal pairs: CD8/Opal 480, CD4/Opal 690, FOXP3/Opal 570, CD68/Opal 520, CD20/Opal 620, and pan-CK/Opal 780 for the first panel. For the second panel, cells were stained with the following primary antibody/conjugated Opal pairs: CD11c/Opal 480, BAP1/Opal 690, LAG3/Opal 570, CD56/Opal 520, MTAP/Opal 620, and NF2/Opal 780. Nuclei were stained with DAPI, and slides were mounted with coverslips using ProLong Diamond Antifade mounting media (Thermo Fisher Scientific). Akoya Bioscience’s PhenoImager HT platform and inForm analysis software were used for whole-slide scanning, spectral unmixing, and phenotyping.

### Analysis of multispectral mIF images

TMA spots were imaged at 20× magnification using a Vectra Polaris system. Subsequently, analysis was performed using Akoya inForm tissue analysis software and an open-source R script called “phenoptr” provided by Akoya. The “phenoptr” script consolidates and analyses output tables generated by inForm.

Within each TMA core, cells were automatically categorized into specific cell types based on marker expression using Akoya’s integrated inForm analysis software. The following surface markers were utilized for cell classification: CD8^+^ for CD8^+^ T cells (cytotoxic T cells), CD4^+^FOXP3^−^ for CD4^+^ T cells, CD4^+^FOXP3^+^ for Tregs, pan-CK^+^ for tumor cells (CK), CD68^+^ for macrophages, CD11c^+^ for DCs, CD56^+^ for NK cells, and CD20^+^ for B cells (Supplementary Table S2).

Measurement matrices, comprising centroid position (*x*, *y*), per-channel intensity, and cell types, were exported for further analysis using R (v4.0.3). In the first round of staining, cells exhibiting positivity for a single marker were assigned to the corresponding cell type. For cells expressing more than one positive marker, an assignment was made based on the marker with the highest intensity, excluding pan-CK. Cells lacking positive markers were categorized as “unidentified”.

Cell-type density was determined using the formula the number of cells of the cell type within the area divided by the total tissue area, expressed as cells/mm^2^. Cell-type proportion was derived from the number of cells of the cell type divided by the total tissue cells. Cell-type intensity is the mean of the all the cell expression of the cell type in the tissue. phenoptr and phenoptrReports were used to identify cellular subsets and perform spatial analyses, including the calculation of cell–cell distances. The distance between two cell subtypes was calculated using the *x* and *y* coordinates derived from the inForm raw data. Per-cell nearest neighbor distances were calculated using phenoptr. This analysis included both cells of the same phenotype and cells of different phenotypes. The mean distance between each cell and its nearest neighbor was automatically determined for both cells.

### Cell-type composition, density, and entropy analysis

We used nine cell-type markers, divided between two rounds of mIF staining. NK (CD56) and DC (CD11c) cell markers were included in the second staining panel, whereas the remaining markers were part of the first panel. In the process of generating combined cell-type compositions, we made adjustments by subtracting the proportions of CD56^+^ and CD11c^+^ cells (number of positive cells/total cells in the core) in the second panel from the proportions of “unidentified” cells in the first panel. This adjustment was made to refine the “unidentified” cell-type values and integrated nine markers in characterization of immune phenotype analysis. Cell-type proportions, reflecting the number of positive cells within a core, were calculated for each core. Subsequently, these proportions were integrated into patient-wise proportions by averaging across all cores associated with a particular patient. Before conducting statistical testing, cell density values were log-transformed. To assess differences in cellular distribution, we compared the proportions of cell types across different types of malignant mesothelioma and within various histologic subtypes within the malignant mesothelioma cases.

To explore both intra- and intertumor heterogeneity, we used the Shannon entropy of cell-type density, utilizing the ChaoShen method implemented in the R “entropy” library (version 1.3.1). Subsequently, we assessed its association with patient survival using the Kaplan–Meier method with the R packages “survminer” (version 0.4.9) and “survival” (version 3.5-8).

### Generation of cell–cell contact matrices

To create cell–cell contact matrices of each pair of cell type, we utilized a custom Java algorithm, which processed FCS files exported from Vortex, initially developed by Goltsev and colleagues (21). The method is based on the Delaunay neighborhood graph to determine cell contacts within the first tier of neighbors. The cell–cell contact score of a pair of cell type was calculated as the log odds ratio (OR) of the observed adjacency frequency compared with the theoretical frequency. The observed probability was derived from the mean of a β-distribution with parameters α (number of connections between cell types A and B) and β (total number of connections – α), whereas the theoretical probability represented the product of the probabilities of occurrence for each cell type. This approach allowed us to account for the contribution of random chance to cell interactions, ensuring that our analysis focused on biologically relevant spatial proximity. The contact score represented the likelihood of direct contact between two phenotypes within the pair, with higher scores indicating a greater likelihood of contact. We rescaled cell contact scores across all phenotype pairs with min–max normalization to the range of [0, 1]. To compare groups, we performed one-sided Mann–Whitney U tests. A FDR value < 0.15 was considered statistically significant.

### Cellular neighborhood analysis

We conducted cellular neighborhood (CN) analysis following previously established methods (22). Each cell was examined in relation to its neighboring cells within a Euclidean distance of 40, 50, and 60 μm from its *xy* coordinates. The neighbors of each cell were represented as a vector, with the length equal to the total number of cell types, containing the frequency of each cell type within its neighboring area. These neighbor vectors for all cells were then integrated and clustered using Python’s scikit-learn library (version 1.3.2) with the MiniBatchKMeans implementation, in which the optimal number of clusters, *K* = 6, was determined using the elbow plot method. Each of these six clusters was designated as a CN, and every cell was assigned to a CN based on its neighbor vector. The CN enrichment score for each cell type was defined as the log OR of the neighborhood cluster centroid to the average frequency of the cell type and was normalized to a range of −5 to 5. Visualization of these scores was achieved using seaborn (version 0.12.2) clustermap.

### Survival analysis

Cox regression univariate analysis was performed using the survival R package based on cell-type proportions and CN scores. Kaplan–Meier curves and log-rank tests were used to assess the association between OS and Shannon entropy of cell-type density. To categorize patients into risk groups, we designated the top 40% of patients as the high-risk group and the bottom 40% as the low-risk group based on the Shannon entropy. We used the log-rank test to compare two groups, testing the null hypothesis that there is no significant difference in OS between the populations. A *P* value <0.05 was considered statistically significant.

### Protein expression association analysis

In our assessment of the association between the protein expression of LAG3, BAP1, NF2, and MTAP and cell-type marker expression, we used Spearman rank correlations to examine their relationships. To investigate cell contacts between immune cells and pan-CK in relation to TSG protein levels, we categorized patients into high and low groups. For example, BAP1-high patients were defined as those falling within the >0.6 quartile, whereas BAP1-low patients were those within the <0.4 quantile of BAP1 density. We considered cores with pan-CK proportions exceeding 20% as tumor enriched. We generated a cell contact heatmap and conducted an analysis comparing MPM and MPeM based on min–max normalized contact scores.

### Statistical analysis and visualization

Statistical analyses of patient characteristics were performed using one-sided Mann–Whitney U tests, with significance set at *P* value < 0.05, a predetermined threshold. We generated graphs using QuPath (23), Phenochart 1.1 with MOTiF (Akoya Biosciences), RColorBrewer (version 1.1 2), ggplot2 (version 3.4.2), gridExtra (version 2.3), viridis (version 0.6.4), ggpubr (version 0.6.0), ggtext (version 0.1.2), ComplexHeatmap (version 2.16.0), ggrepel (version 0.9.1), and ggridges (version 0.5.4) packages. For general data analysis and manipulation, we utilized the following packages and tools: stats (version 4.3.1), survival (version 3.5-8), survminer (version 0.4.9), coin (version 1.4-2), tidyverse (version 2.0.0), dplyr (version 1.1.2), data.table (version 1.14.8), entropy (version 1.3.1), and xlsx (version 0.6.5) within R (version 4.3.1). Additionally, we used NumPy (version 1.21.5), pandas (version 1.4.4), and SciPy (version 1.7.3) with Python (version 3.9).

### Web portal for data access and visualization

We developed a user-friendly web portal (https://mesotheliomaspatialatlas.streamlit.app/) to facilitate access and visualization of mIF and H&E images. This portal allows users to search, view, and download all core images without registration or log in. To obtain high resolution of individual core image, we utilized the segment_anything (version 1.0.00) package to segment cores on an image file (.png) converted from whole-scan image (.sys file) using OpenSlide Python (version 1.2.0). The web portal was constructed with a Python (version 3.11) backend and implemented using the Streamlit (version: 1.25.0) open-source framework. The application was deployed on the Streamlit cloud platform and has been tested for compatibility with Google Chrome and Apple Safari browsers. Static figures and logos used within the portal were created using BioRender (www.biorender.com).

### Data availability

The H&E and mIF images are available at https://github.com/osmanbeyoglulab/MesotheliomaSpatialAtlas_data. Initial input data for the analysis are available at https://sites.pitt.edu/∼xim33/Mesothelioma. The remaining data are available within the article and supplementary information.

### Code availability

The code for the web resource is available at https://github.com/osmanbeyoglulab/MesotheliomaSpatialAtlas_webapp. The code for the data analysis is available at https://github.com/osmanbeyoglulab/MesotheliomaSpatialAtlas_analysis.

## Results

### Dataset and patient characteristics

**Figure 1:**
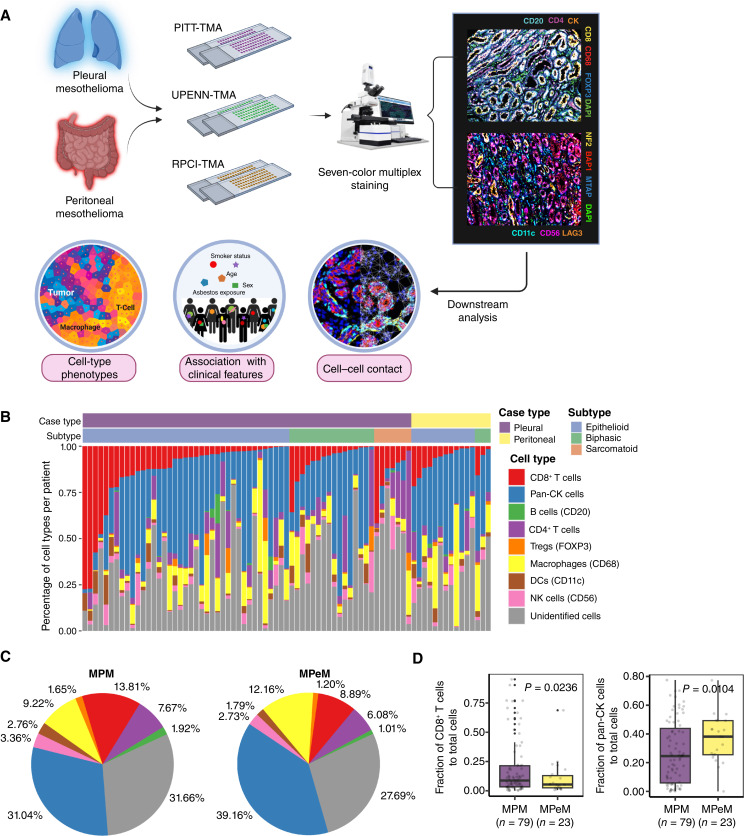
Profiling the spatial landscape of malignant mesothelioma using mIF. **A,** Schematic diagram outlining the acquisition of multiplexed immunofluorescence images from patients with malignant mesothelioma generated using BioRender.com. **B,** Waterfall plot depicting the distribution of cell populations as a percentage of all cells in the TIME, categorized by CD8^+^ T cells and sorted across histologic subgroups within MPM and MPeM. Vertical bars represent cell percentages, with distinct colors corresponding to various cell lineages. **C,** Pie charts depicting the mean cellular composition in samples with MPM and MPeM, including relative frequencies of B cells, CD4^+^ T cells, CD8^+^ T cells, Tregs, macrophages, DCs, NK cells, pan-CK^+^ tumor cells, and unidentified cells. **D,** Assessment of the prevalence of CD8^+^ T cells and pan-CK^+^ tumor cells in MPM and MPeM as a fraction of the total cell population. *P* values were calculated using the one-sided Mann–Whitney U test. RPCI, Roswell Park Cancer Institute; UPENN, University of Pennsylvania; UPITT, University of Pittsburgh.

To characterize the spatial distribution of cellular components within the malignant mesothelioma TIME, we used mIF analysis on tissue samples obtained from patients diagnosed with MPM and MPeM (Fig. 1A). Our dataset comprised three TMAs (core size = 0.6 mm) containing 336 cores from 115 patients, one sample per patient. Each patient contributed between 1 and 9 cores, with 13 patients contributing only 1 core, and 80% of patients (*n* = 80) were represented by more than three cores. These TMAs were obtained from the NMVB. Patients received care at Roswell Park Cancer Institute (*n* = 53), University of Pennsylvania (*n* = 30), or University of Pittsburgh/University of Pittsburgh Medical Center (*n* = 32). Detailed information about cohort characteristics and associated clinicopathologic features is described in Table 1.

**Table 1:** Characteristics of patients with malignant mesothelioma

Characteristic	Total *n* = 115	UPENN *n* = 30	UPITT *n* = 32	RPCI *n* = 53
Sex, *n* (%)				
Female	24 (21%)	3 (10%)	7 (22%)	14 (26%)
Male	81 (70%)	18 (60%)	24 (75%)	39 (74%)
Unknown	10 (8.7%)	9 (30%)	1 (3.1%)	0 (0%)
Race, *n* (%)				
American Indian and Aleutian Eskimo	1 (0.9%)	0 (0%)	0 (0%)	1 (1.9%)
Asian	1 (0.9%)	0 (0%)	0 (0%)	1 (1.9%)
Black	2 (1.7%)	0 (0%)	1 (3.1%)	1 (1.9%)
White	93 (81%)	18 (60%)	25 (78%)	50 (94%)
Unknown	18 (16%)	12 (40%)	6 (19%)	0 (0%)
Diagnosis age, *n* (%)				
31–40 years	4 (3.5%)	0 (0%)	3 (9.4%)	1 (1.9%)
41–50 years	8 (7.0%)	3 (10%)	3 (9.4%)	2 (3.8%)
51–60 years	25 (22%)	7 (23%)	6 (19%)	12 (23%)
61–70 years	47 (41%)	14 (47%)	14 (44%)	19 (36%)
71–80 years	27 (23%)	6 (20%)	5 (16%)	16 (30%)
81–90 years	4 (3.5%)	0 (0%)	1 (3.1%)	3 (5.7%)
Asbestos exposure, *n* (%)				
No	6 (5.2%)	0 (0%)	0 (0%)	6 (11%)
Yes	38 (33%)	7 (23%)	12 (38%)	19 (36%)
Unknown	71 (62%)	23 (77%)	20 (62%)	28 (53%)
Smoking status, *n* (%)				
Current smoker	13 (11%)	0 (0%)	3 (9.4%)	10 (19%)
Nonsmoker	33 (29%)	2 (6.7%)	14 (44%)	17 (32%)
Previous smoker	36 (31%)	5 (17%)	5 (16%)	26 (49%)
Smoker (current or previous)	2 (1.7%)	2 (6.7%)	0 (0%)	0 (0%)
Not available	31 (27%)	21 (70%)	10 (31%)	0 (0%)
Case type, *n* (%)				
Peritoneal (MPeM)	25 (22%)	3 (10%)	10 (31%)	12 (23%)
Pleural (MPM)	88 (77%)	27 (90%)	20 (62%)	41 (77%)
Other	2 (1.7%)	0 (0%)	2 (6.2%)	0 (0%)
Histology, *n* (%)				
Benign fibrous	2 (1.7%)	0 (0%)	2 (6.2%)	0 (0%)
Biphasic	22 (19%)	4 (13%)	8 (25%)	10 (19%)
Desmoplastic	1 (0.9%)	0 (0%)	1 (3.1%)	0 (0%)
Epithelioid	69 (60%)	22 (73%)	17 (53%)	30 (57%)
Fibrocystic	1 (0.9%)	0 (0%)	1 (3.1%)	0 (0%)
Multicystic	1 (0.9%)	0 (0%)	0 (0%)	1 (1.9%)
Papillary	3 (2.6%)	0 (0%)	2 (6.2%)	1 (1.9%)
Sarcomatoid	8 (7.0%)	4 (13%)	1 (3.1%)	3 (5.7%)
Not specified	8 (7.0%)	0 (0%)	0 (0%)	8 (15%)
Grade, *n* (%)				
High	46 (40%)	11 (37%)	27 (84%)	8 (15%)
Intermediate	17 (15%)	15 (50%)	2 (6.2%)	0 (0%)
Low	8 (7.0%)	4 (13%)	3 (9.4%)	1 (1.9%)
Not specified	44 (38%)	0 (0%)	0 (0%)	44 (83%)
Survival period	12 (7, 24)	12 (9, 16)	7 (3, 24)	15 (8, 29)

Abbreviations: RPCI, Roswell Park Cancer Institute; UPENN, University of Pennsylvania; UPITT, University of Pittsburgh.

Our integrated cohort (*n* = 115) was predominantly male (70%), with 21% being female, and sex information for the remaining cases was not specified. The cohort contained primarily White patients (81%). The median age at diagnosis was 61 to 70 years, constituting 41% of patients. Asbestos exposure was confirmed in only 33% of cases, though asbestos exposure was unknown in the majority of cases (62%). Moreover, 44% of patients had a documented history of smoking; however, data were unavailable for 27% of the cases.

The majority of cases (77%) were diagnosed as MPM, with a smaller proportion (22%) categorized as MPeM. The remaining cases constituted various other types of malignant mesothelioma. Histologically, epithelioid tumors were the most prevalent (60%) among our patient cohort, whereas 19% of the cases were biphasic. Additionally, there were smaller representations of other histologic types, such as sarcomatoid, papillary, and benign fibrous. Notably, 7% of cases fell under the category of unspecified histology. The median survival period from the time of diagnosis for our cohort was 12 months, with an IQR of 7 to 24 months. The patients with MPeM generally exhibited a significantly higher OS than their MPM counterparts (log-rank *P* = 0.005). Furthermore, patients with no asbestos exposure demonstrated a significantly higher OS (*P* = 0.015).

### Characterization of immune cell phenotypes in malignant mesothelioma

The TIME represents a dynamic ecosystem where intricate spatial interactions occur among tumor cells, stromal components, and immune cells. To gain further insights into the spatial distribution of key immune cell populations in MPM and MPeM, we performed mIF on TMA sections from malignant mesothelioma samples using two Opal seven-color IHC panels (Fig. 1A). The first panel featured staining for tumor cells (pan-CK^+^), B cells (CD20^+^), CD4^+^ and CD8^+^ T cells, Tregs (CD4^+^FOXP3^+^), and macrophages (CD68^+^). In the second panel, we included the identification of DC cells (CD11c^+^) and NK cells (CD56^+^), as well as the assessment of certain proteins, namely, LAG3, BAP1, NF2, and MTAP. In addition to the immune checkpoint LAG3, BAP1, NF2, and MTAP were chosen as proxies to represent commonly altered TSG products in malignant mesothelioma. The images and data undergo a series of tissue and cell quality checks. To facilitate comparisons across different histologic and pathologic groups of MPM and MPeM, we performed cell-type quantification. The composition of the TMAs, the tumor fractions, and percentage/density/intensity of each marker per core used in downstream analysis are shown in Supplementary Table S2.

We conducted an analysis of cell-type density correlation both within cores from the same patient and across different patients. Our findings revealed a higher correlation between cores sampled from the same patient, suggesting a consistent level of intratumor heterogeneity. Conversely, cores from different patients exhibited a lower correlation, indicating a higher degree of intertumor diversity (average Pearson correlation of 0.7403 and 0.5178 within and between patients, respectively; *P* = 0.5 × 10^−30^; Supplementary Fig. S2A). Additionally, we used Shannon entropy to quantify intratumor heterogeneity within malignant mesothelioma lesions. This method enabled us to assess the diversity of tumor and immune cell populations across patient samples. Patients with a minimum of two cores were included in this analysis, and the average Shannon entropy was computed for each patient. Subsequently, patients were divided into high and low heterogeneity groups based on Shannon entropy quantiles (>0.6 and <0.4, respectively). Statistical analysis using the Kaplan–Meier method revealed a significant difference in OS between these groups, with a *P* value of 0.03 (see Supplementary Fig. S2B). Interestingly, the group with higher intratumor heterogeneity (Shannon entropy > 0.6 quantile) showed a favorable association with OS.

The predominant immune cell populations comprised lymphocytes (CD8^+^ and CD4^+^ T cells) and CD68^+^ macrophages, whereas B cells and Tregs were relatively less abundant (Fig. 1B). An evaluation of the overall immune composition of MPM and MPeM revealed a slightly higher proportion of CD8^+^ T cells in MPM (13.81%) than MPeM (8.89%). Conversely, the relative contribution of tumor cells (pan-CK^+^) was higher in MPeM (39.16%) than that in in MPM (31.04%) samples (Fig. 1C and D). These distinctions did not reach statistical significance because of substantial variability among patients within the same tumor types.

**Figure 2:**
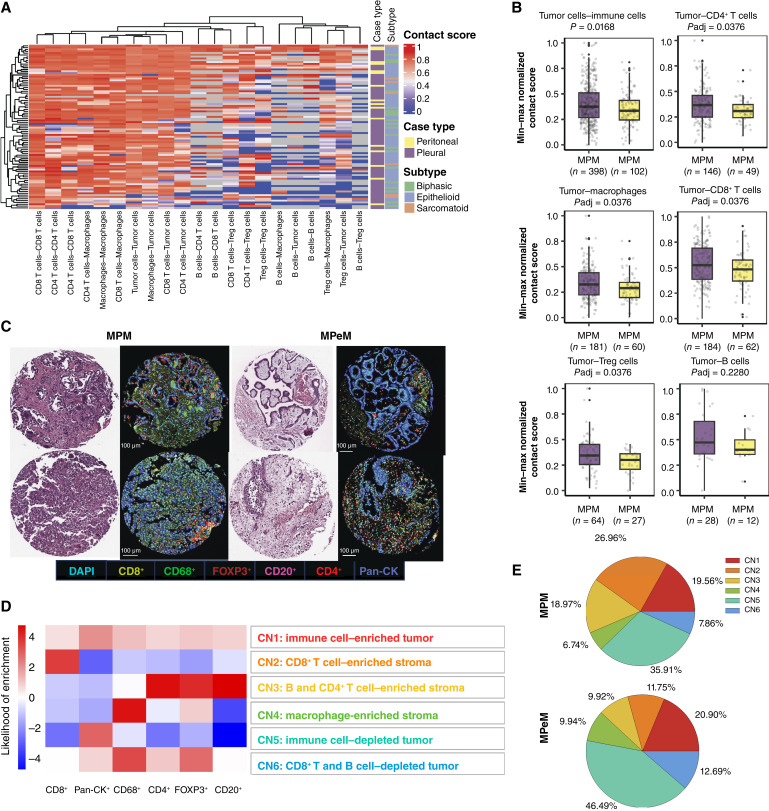
Interactions between immune cells and tumor cells in malignant mesothelioma. **A,** Heatmap depicting cell–cell contacts in patients with MPM or MPeM. Columns represent normalized pairwise cell–cell contact scores, and rows represent samples. **B,** Comparison of contact scores between tumor cells and the overall immune cell population and contact scores specifically involving tumor cells and CD4^+^ T cells, CD8^+^ T cells, macrophages, and Tregs between cohorts of patients with MPM and MPeM. Statistical analysis was performed using FDR-adjusted one-sided multiple Mann–Whitney U tests. Each dot represents the cell contact score of an individual core. **C,** Representative cases from two samples with MPM and two patients with MPeM demonstrating mIF, H&E staining, and cell-type analyses. Images were captured at 10× magnification. Scale bar, 100 μm. **D,** Heatmap depicting types of CNs (rows) and relative enrichment above or below the mean across neighborhoods for B cells (CD20^+^), CD8^+^ T cells, CD4^+^ T cells, Tregs (FOXP3^+^), tumor cells (Pan-CK^+^), and macrophages (CD68^+^). Likelihood of enrichment was calculated as log odds ratio values normalized between 5 and −5. **E,** Pie charts representing median relative contributions of CNs in MPM (top) and MPeM (bottom) biologically independent samples.

In addition to cell-type proportions within the malignant mesothelioma TIME, the spatial organization of these cells offers valuable insights into their roles in malignant mesothelioma development, progression, and responses to therapy. Consequently, we computed pairwise cell contact scores for the first panel, which included the tumor CK marker (pan-CK^+^; Fig. 2A). Although the overall distribution of immune cells within the TIME exhibited similarities between MPM and MPeM, we observed heightened interactions between immune cells and tumor cells in MPM relative to MPeM. This encompassed interactions between tumor cells and different immune cells, such as CD4^+^ T cells, CD8^+^ T cells, macrophages, and Tregs (FDR < 0.1, Fig. 2B and C; Supplementary Table S3). Such increased tumor–immune cell interactions may influence the immune responses against MPM and MPeM and contribute to the divergent behaviors observed in these two forms.

To explore the impact of spontaneously organized, spatially resolved clusters of cells within the malignant mesothelioma tissue architecture, we conducted CN analysis as described previously (22). Through unbiased clustering and nearest neighbor analysis, we identified six distinct CNs within the malignant mesothelioma TIME (Fig. 2D): CN1 (immune cell–enriched tumor), CN2 (CD8^+^ T cell–enriched stroma), CN3 (B and CD4^+^ T cell–enriched stroma), CN4 (macrophage-enriched stroma), CN5 (immune cell–depleted tumor), and CN6 (B and CD8^+^ T cell–depleted tumor). Subsequently, we evaluated the prevalence of these neighborhoods across MPM and MPeM TIMEs, revealing higher percentages of CN2 and CN3 in MPM than MPeM and higher percentages of CN4 and CN6 in MPeM than MPM (Fig. 2E). The expansion of CN2 and CN3 in MPM suggests a greater potential for an adaptive immune response in this subtype. To mitigate potential biases stemming from a fixed distance threshold for neighborhood definition, we conducted similar analyses using additional thresholds of 40 and 60 μm. Notably, our findings across MPM and MPeM remained consistent across all three distance measures. Although there were some fluctuations in the odds of enrichment within neighborhoods, the compositions of neighborhoods, especially those significantly different between MPM and MPeM, remained consistent across the 40, 50, and 60 μm thresholds. These findings enhance the robustness and reliability of our observations.

### Immune cell phenotypes and CNs associated with clinical features and survival outcomes

To investigate the relationship between immune cell populations and clinical or pathologic variables, we analyzed the frequency of individual cell types as a percentage of total cells per patient. We correlated the imaging data with various clinical parameters, including histology, survival, sex, age, asbestos exposure, smoking status, and tumor grade. Although the current histologic classification of malignant mesothelioma holds a prognostic value, it is important to note that significant variability exists in clinical features and patient outcomes even within these histologic subtypes.

**Figure 3:**
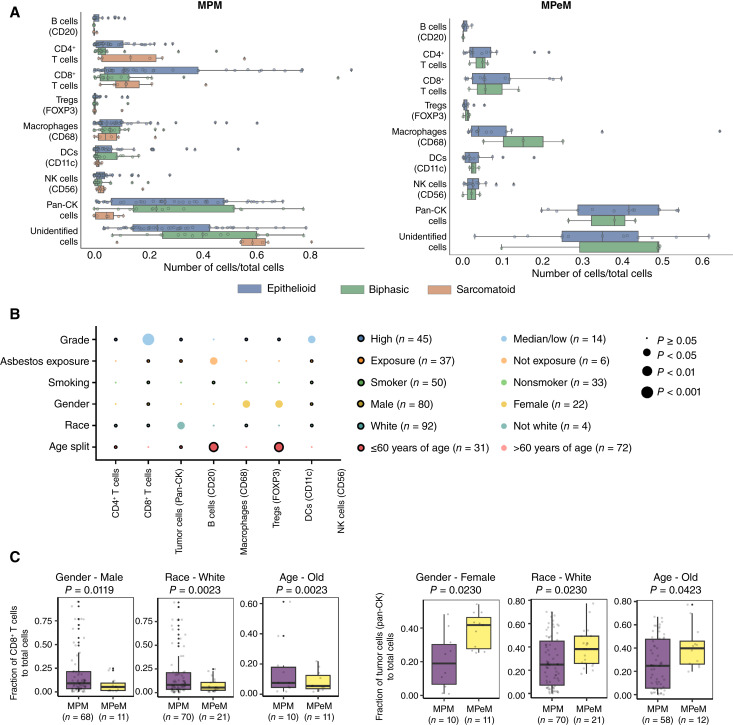
Immune cell phenotypes associated with clinical features. **A,** Prevalence of all cell types in MPM epithelioid (*n* = 45), biphasic (*n* = 16), and sarcomatoid (*n* = 7) subtypes (left) and MPeM epithelioid (*n* = 12) and biphasic (*n* = 3) subtypes (right) presented as the proportion of total cells. Statistical analysis was performed using one-sided Multiple Mann–Whitney nonparametric U tests. *, *P* < 0.05. **B,** Bubble plot illustrating significance levels, in which circle size corresponds to the level of significance, and circle color indicates which of the two comparisons on the *y*-axis shows higher levels of the cell type plotted on the *x*-axis. **C,** Box plots displaying cell-type proportion across different clinical subgroups in patients with MPM and MPeM. Statistical analysis was conducted using one-sided multiple Mann–Whitney U tests. *P* < 0.05 was considered significant. The center of the box plot represents the median, with the box boundaries indicating the 25th and 75th percentiles. Whiskers extend to the minimum and maximum values in the dataset.

Our analysis revealed that immune cell proportions among different histologic groups were generally similar (Fig. 3A; Supplementary Table S4). Notable differences included higher proportions of B cells in epithelioid MPM tumors than biphasic tumors, higher proportions of CD4^+^ T cells in epithelioid and sarcomatoid MPM tumors than biphasic tumors, and greater amounts of pan-CK^+^ cells in biphasic MPMs than sarcomatoid tumors (*P* < 0.05; Supplementary Table S4). However, some variations in cell-type proportions did not reach statistical significance because of the inherent variability in cell frequencies across specimens. Moreover, when we compared epithelioid/biphasic MPM and MPeM, we did not find statistically significant differences in cell compositions (Supplementary Table S5).

We conducted an exploratory survival analysis to investigate the relationships between immune cell infiltration, CNs, and clinical outcomes. Our findings revealed that an increase in CD4^+^ T cells (log-rank *P* = 0.01) and CN3 (B and CD4^+^ T cell–enriched stroma; log-rank *P* = 0.01) percentages was correlated with improved survival outcomes in patients with MPeM (log-rank *P* = 0.03). Conversely, CN3 (B and CD4^+^ T cell–enriched stroma) was associated with worse survival in MPM (*P* = 0.01; Supplementary Table S6).

Although we did not identify survival associations for several immune cell types and CNs, we did uncover relationships between cell frequencies and specific clinical subgroups (Fig. 3B). Patients with median/low-grade tumors exhibited higher proportions of CD8^+^ T cells and DCs than those with high-grade tumors. Patients without asbestos exposure exhibited higher proportions of B cells, whereas female patients had higher proportions of macrophages and Tregs. Younger patients demonstrated higher proportions of Tregs and B cells. No significant associations were found between smoking status and immune cell infiltration. Furthermore, when we compared patients with MPM and MPeM based on gender, race, and age, we observed statistically significant differences in CD8^+^ T and pan-CK^+^ proportions (Fig. 3C; Supplementary Table S7). Collectively, these findings suggest that understanding the spatial organization of the TIME may provide valuable insights into individual patient survival outcomes beyond traditional histologic subtype classifications and individual cell prevalence.

### Malignant mesothelioma architecture and TSG protein expression associations

**Figure 4:**
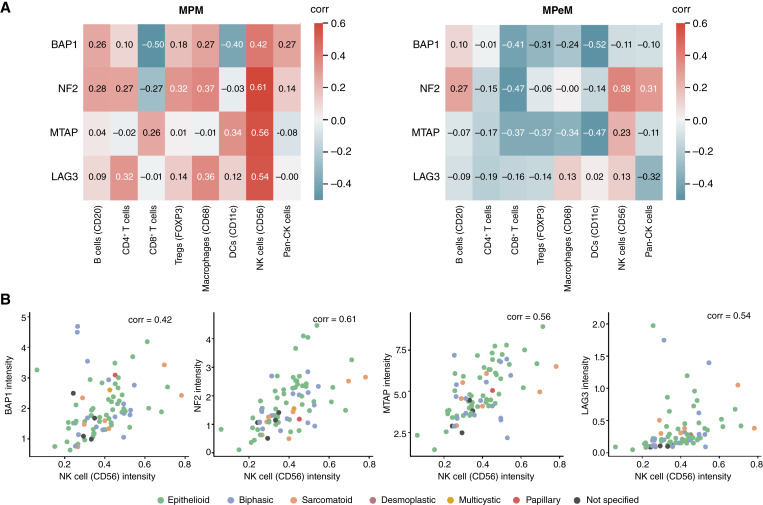
Association of BAP1, NF2, MTAP, and LAG3 intensities with immune cell–type intensity in MPM or MPeM. **A,** Heatmaps showing correlations between the mean intensity of immune cell markers and expression levels of BAP1, NF2, MTAP, and LAG3 across biologically independent tumors in MPM and MPeM. **B,** Correlation between mean CD56 (NK cell) intensity and mean BAP1, NF2, MTAP, and LAG3 expression. Dots are color-coded by histologic subtypes.

We next investigated the influence of the protein expression of BAP1, NF2, CDKN2A, and LAG3 on the malignant mesothelioma TIME. A prior study using a monoclonal anti-MTAP primary antibody reported acceptable specificity and sensitivity for MTAP immunohistochemistry in detecting the CDKN2A homozygous deletion and diagnosis of malignant mesothelioma (24). In both MPM and MPeM, we observed that the average expression of the BAP1 protein was negatively correlated with CD8 and CD11c protein expression (Fig. 4A), with a Spearman correlation coefficient (ρ) ≤ −0.4. CD8^+^ T cells directly target infected or cancerous cells, whereas DCs play a role in antigen presentation to other immune cells. Further analysis of individual spots revealed a significant positive correlation between tumor infiltration by NK cells (CD56^+^) and average BAP1, NF2, MTAP, and LAG3 expression levels in MPM (ρ > 0.4; Fig. 4B).

**Figure 5:**
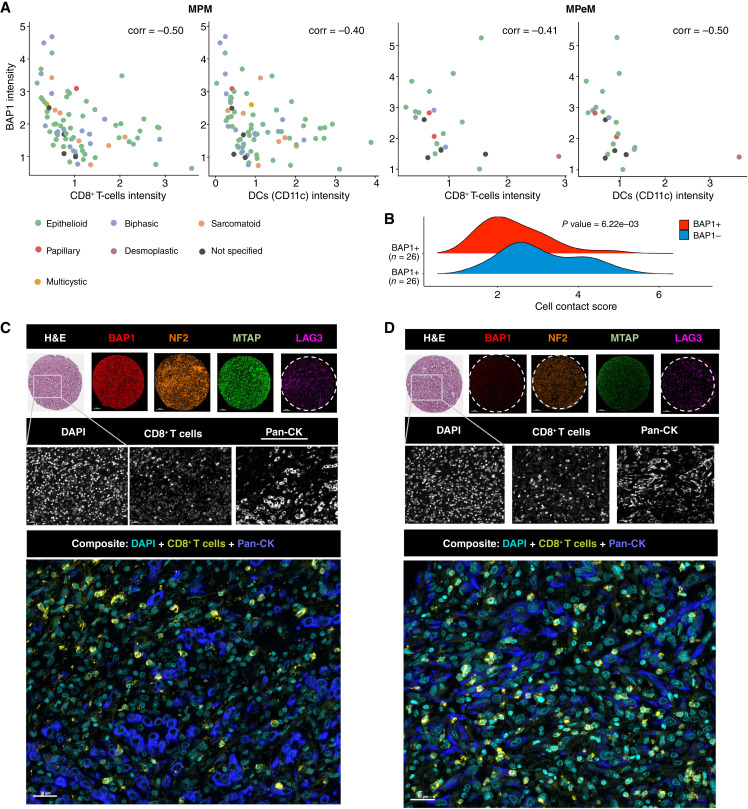
Analysis of cell types in MPM BAP1-high and BAP1-low tumor groups. **A,** Correlation between mean CD8^+^ (cytotoxic T-cell) and CD11c^+^ (DC) intensities and mean BAP1 expression levels. Dots are color-coded by histologic subtypes. **B,** Ridge density plot illustrating tumor and CD8^+^ T cell contact scores among BAP1-high and BAP1-low pan-CK–enriched cores (cores with CK percent>20%). Density values >0.6 quantile and <0.4 quantile were used as thresholds to categorize cores as BAP1-high and BAP1-low, respectively. **C** and **D,** Segmented images show increased interaction between cancer cells and CD8^+^ T cells in BAP1-low MPM (**D**) compared with BAP1-high MPM (**C**). Top, TMA core sections stained with H&E, in which BAP1 is represented in Opal 690 (red); NF2 in Opal 780 (orange); MTAP in Opal 620 (green); and LAG3 in Opal 570 (magenta). Scale bars, 100 μm, 10× original magnification. Middle, Zoomed-in area from the boxed segment in the top, stained with DAPI for DNA (cyan); Opal 480 for CD8^+^ T cells (yellow); and Opal 780 for pan-CK cells (blue). Scale bars, 10 μm, 100× original magnification. Bottom, Composite images. Scale bars, 30 μm, 30× original magnification.

We observed significantly greater tumor infiltration by cytotoxic T cells and DCs in MPM and MPeM tumors with BAP1-low expression (Fig. 5A). To further investigate the relationship between BAP1 expression and the TIME, we assessed pairwise contacts between various immune cell types and tumor cells from malignant mesothelioma tumors with BAP1-high and BAP1-low expression levels. Analysis of pairwise contacts in tumor samples revealed that the BAP1-low group was enriched with pan-CK–CD8^+^ T-cell contacts in MPM but not in MPeM cases (Fig. 5B and C; Supplementary Table S8). This observation holds potential implications for understanding the immune responses of and devising therapeutic strategies for patients with MPM characterized by reduced BAP1 expression in tumor cells.

### The Human Spatial Atlas of Malignant Mesothelioma

The Human Spatial Atlas of Malignant Mesothelioma, available at https://mesotheliomaspatialatlas.streamlit.app/, is a valuable web resource we have developed for researchers and scientists. This resource offers interactive visualizations of our mIF dataset and H&E images for each core within the TMAs. Furthermore, it facilitates an in-depth exploration of the spatial aspects of the malignant mesothelioma TIME, providing a unique opportunity to study malignant mesothelioma pathogenesis and potentially identify new avenues for therapy development.

## Discussion

We utilized mIF technology to conduct a comprehensive analysis of malignant mesothelioma TMAs, offering a detailed spatial comparison of the malignant mesothelioma TIME between MPeM and MPM. This represents the most extensive mIF examination of malignant mesothelioma samples since Parra and colleagues’ initial study with 12 malignant mesothelioma cases (25). Additionally, we introduce The Human Spatial Atlas of Malignant Mesothelioma (https://mesotheliomaspatialatlas.streamlit.app/), an online resource housing our mIF data and H&E images. This interactive platform is designed to serve researchers, providing insights into the malignant mesothelioma TIME by providing visualizations for each core within the TMAs. Beyond our research, this resource serves as an invaluable tool for testing hypotheses related to the malignant mesothelioma TIME and unraveling the intricacies of malignant mesothelioma pathogenesis. Considering the limited therapeutic options available to patients with malignant mesothelioma, there is substantial translational potential in understanding the correlation between the spatial architecture of the malignant mesothelioma TIME and tumor biology. Moreover, it raises questions about harnessing specific immune cell subsets to enhance clinical outcomes in this lethal disease.

In our pursuit of a better understanding of malignant mesothelioma, we delved into the intricacies of the immune cell landscape within MPM and MPeM. Our analysis revealed a consistent pattern, with T cells and macrophages significantly enriched in both malignant mesothelioma tumor tissues, regardless of tumor site of origin. Although the MPM TIME slightly favored T cells, the MPeM TIME leaned more toward macrophages. To explore the potential cross-talk between these immune cells and tumor cells, we used cell–cell contact analysis. Interestingly, we found that the malignant mesothelioma TIME shares remarkable similarities between MPM and MPeM, with one notable exception: MPM tumors exhibited significantly more interactions between immune cells and tumor cells. Despite the prevalence of T cells, recent T cell–centric therapies, such as the CheckMate 743 study for MPM, have shown only modest responses (7, 8). Yin and colleagues recently demonstrated the significance of the high density and spatial proximity of CD8^+^ T cells to tumor cells, demonstrating their correlation with improved responses to nivolumab, whereas the close proximity of Tregs to tumor cells correlated with poorer responses (26). Our findings suggest that the distinct TIME landscape may serve as a predictive factor for the efficacy of immune checkpoint inhibitors in MPM.

Understanding the relationship between TSG expression and immune cell presence and function is crucial for shaping effective immunotherapy strategies. If a tumor’s TSG status influences immune cell infiltration and activity, it could provide valuable insights for developing treatments that modulate the TIME, ultimately enhancing T/NK/DC cell–mediated tumor clearance. Through our mIF analyses, we found that BAP1-low tumors exhibit elevated infiltration by CD8^+^ T cells and DCs. Consequently, BAP1 expression emerges as a promising biomarker for T cell–centric therapies. However, our study unveils nuanced distinctions between the malignant mesothelioma types. MPM may possess a superior ability to trigger an adaptive immune response compared with MPeM. Interestingly, in MPeM, but not in MPM, we observed an inverse correlation between BAP1 expression and the intensity of immune cell markers for CD8^+^ T cells, Tregs, macrophages, and DCs (Fig. 4A). For MPM, this inverse correlation was only apparent with markers for CD8^+^ T cells and DCs. This suggests that in MPeM, the loss of BAP1 may correlate with tumor infiltration by various immune cell types, including CD8^+^ T cells, Tregs, macrophages, and DCs. In contrast, in MPM, increased infiltration is observed only with CD8^+^ T cells and DCs when *BAP1* alone is deleted. Whether these alterations in the spatial landscape of the malignant mesothelioma TIME contribute, at least partially, to the generally more favorable prognosis of MPM than MPeM, or the improved survival prospects of patients with *BAP1* alterations alone among MPM cases, remains a topic for future research.

### Limitations

Our analysis relies on staining small biopsy specimens within the TMAs, which may not fully represent the entire tumor. This limitation is particularly relevant given the evidence of intratumor heterogeneity and polyclonality in malignant mesothelioma (27, 28). However, the majority of our tumors are represented by multiple cores, enhancing the comprehensiveness of our dataset. Second, the relatively small sample size imposes constraints on statistical power and the flexibility of multivariable modeling. Although acknowledging this limitation, it is important to underscore that malignant mesothelioma is a rare cancer, and our study represents the largest mIF study based on malignant mesothelioma TMAs to date. Additionally, our study is limited by nine markers, which restricts our ability to distinguish between specific immune cell subtypes. Specifically, we cannot discern exhausted versus effector CD8^+^ T cells or differentiate between tumor-associated and inflammatory macrophages, despite the known influence of these subtypes on patient outcomes (9). Third, our study lacks matching clinical data about responses to treatment. This limitation arises from the retrospective nature of our analysis.

## Conclusions

Our study has the potential to establish TIME architecture as a novel marker in malignant mesothelioma. It contributes to a rapidly expanding body of literature that emphasizes the significance of spatially resolved datasets in elucidating the relationship between TIME architecture and malignant mesothelioma biology.

## Supplementary Material

Supplementary Table 2 Clinical features, cell count, tumor percentage, and density associated with each core, including marker percentage, density and intensity for each core.

Supplementary Figure 1 Example of 7-plex healthy donor lymphoid tissue as a control and for validation. All channels combined as well as Individual marker channels (pCK: pan-cytokeratin).

Supplementary Figure 2 MM intra-tumor heterogeneity and survival analysis. (A) Comparison of TMA core cell-type proportions within patients and across patients. Each value in the boxplot represents the Pearson correlation coefficients between cell-type proportions across cores from different patients and cores within individual patients. (B) Kaplan-Meier survival curves of MM patients that categorized into two groups based on intra-tumor heterogeneity: those with high intra-tumor heterogeneity (Shannon entropy > 0.6 quantile) shown in blue, and those with low intra-tumor heterogeneity (Shannon entropy < 0.4 quantile) depicted in yellow.

Supplementary Table 1 Dataset characteristics

Supplementary Table 3 Comparison of differences in cell–cell contact scores between malignant pleural mesothelioma (MPM) and malignant peritoneal mesothelioma (MPeM) tumors.

Supplementary Table 4 Distribution of immune cells and tumor cells across different histologies in the malignant mesothelioma (MM) cohort.

Supplementary Table 5 Comparison of cell type distributions between malignant pleural mesothelioma (MPM) and malignant peritoneal mesothelioma (MPeM) within the same histologic subtypes.

Supplementary Table 6 The association of immune cells and cellular neighborhood proportion with overall survival in all malignant mesothelioma (MM) as well as for malignant pleural mesothelioma (MPM) and malignant peritoneal mesothelioma (MPeM) separately.

Supplementary Table 7 Comparison of clinical parameters between malignant pleural mesothelioma (MPM) and malignant peritoneal mesothelioma (MPeM) for various cell types.

Supplementary Table 8 Comparison of cell–cell contact score differences between malignant pleural mesothelioma (MPM) and malignant peritoneal mesothelioma (MPeM).
