# Supplementary material for: Spatial Landscape of Malignant Pleural and Peritoneal Mesothelioma Tumor Immune Microenvironments
Source: Cancer Res Commun. 2024 Aug 16;4(8):2133–46. doi: 10.1158/2767-9764.CRC-23-0524 (PMC11328914; doi:10.1158/2767-9764.CRC-23-0524)
Supplement: Supplementary Figure 1 — Example of 7-plex healthy donor lymphoid tissue as a control and for validation. All channels combined as well as Individual marker channels (pCK: pan-cytokeratin). [file crc-23-0524_supplementary_figure_1_suppsf1.docx]

**Supplementary Figure 1**


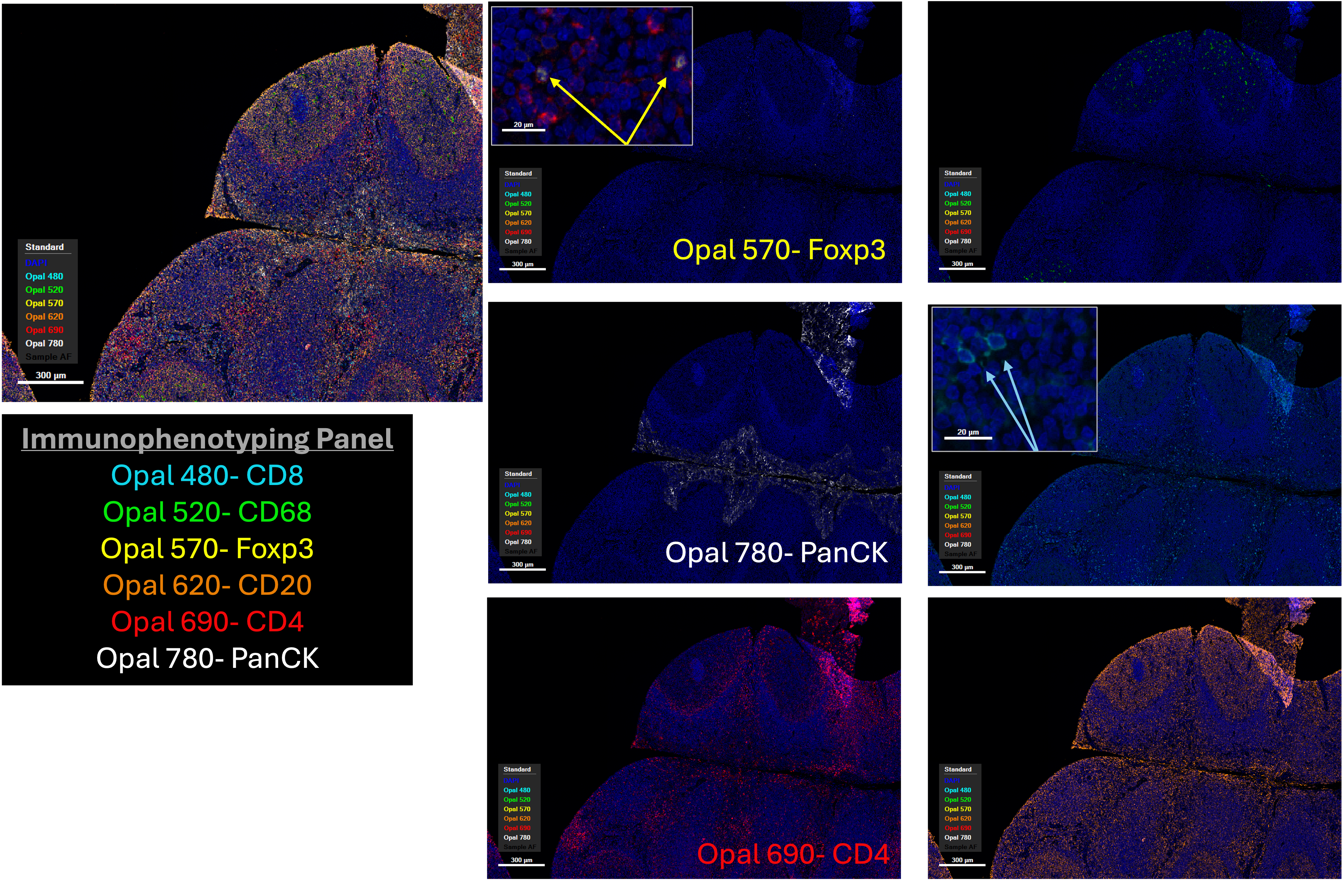


**Supplementary Figure 1**

Supplementary Figure shows example of 7-plex healthy donor lymphoid tissue as a control and for validation. All channels combined as well as Individual marker channels (pCK: pan-cytokeratin).
