# Supplementary material for: Spatial Landscape of Malignant Pleural and Peritoneal Mesothelioma Tumor Immune Microenvironments
Source: Cancer Res Commun. 2024 Aug 16;4(8):2133–46. doi: 10.1158/2767-9764.CRC-23-0524 (PMC11328914; doi:10.1158/2767-9764.CRC-23-0524)
Supplement: Supplementary Figure 2 — MM intra-tumor heterogeneity and survival analysis. (A) Comparison of TMA core cell-type proportions within patients and across patients. Each value in the boxplot represents the Pearson correlation coefficients between cell-type proportions across cores from different patients and cores within individual patients. (B) Kaplan-Meier survival curves of MM patients that categorized into two groups based on intra-tumor heterogeneity: those with high intra-tumor heterogeneity (Shannon entropy > 0.6 quantile) shown in blue, and those with low intra-tumor heterogeneity (Shannon entropy < 0.4 quantile) depicted in yellow. [file crc-23-0524_supplementary_figure_2_suppsf2.docx]

**Supplementary Figure 2**

**Supplementary Figure 2**

Supplementary Figure 2 shows MM intra-tumor heterogeneity and survival analysis. **(A)** Comparison of TMA core cell-type proportions within patients and across patients. Each value in the boxplot represents the Pearson correlation coefficients between cell-type proportions across cores from different patients and cores within individual patients. **(B)** Kaplan-Meier survival curves of MM patients that categorized into two groups based on intra-tumor heterogeneity: those with high intra-tumor heterogeneity (Shannon entropy > 0.6 quantile) shown in blue, and those with low intra-tumor heterogeneity (Shannon entropy < 0.4 quantile) depicted in yellow.
