# Supplementary material for: Spatial Landscape of Malignant Pleural and Peritoneal Mesothelioma Tumor Immune Microenvironments
Source: Cancer Res Commun. 2024 Aug 16;4(8):2133–46. doi: 10.1158/2767-9764.CRC-23-0524 (PMC11328914; doi:10.1158/2767-9764.CRC-23-0524)
Supplement: Supplementary Table 1 — Dataset characteristics [file crc-23-0524_supplementary_table_1_suppst1.docx]

**Supplementary Table 1: Dataset characteristics**

|  | **UPenn** | **Pitt** | **RPCI** | **TOTAL** |
| --- | --- | --- | --- | --- |
| **Number of TMA slides** | 1 | 1 | 1 | 3 |
| **Core diameter** | 0.6 mm | 0.6 mm | 0.6 mm | - |
| **Number of cores** | 103 | 72 | 161 | 336 |
| **Number of valid cores** | 77 | 66 | 161 | 304 |
| **Number of cases** | 30 | 32 | 53 | 115 |
| **Number of cases with valid cores** | 22 | 29 | 53 | 104 |
| **Number of cores per case** | 1 (1 case) 2 (8 cases) 3 (6 cases) 5 (3 cases) 6 (3 cases) 9 (1 case) | 1 (12 cases) 2 (3 cases) 3 (8 cases) 4 (6 cases) | 2 (1 case) 3 (51 cases) 6 (1 case) | 1 (13 cases) 2 (12 cases) 3 (65 cases) 4 (6 cases) 5 (3 cases) 6 (4 cases) 9 (1 case) |
| **Number of cells** | 163,488 | 129,185 | 283,359 | 576,032 |
| **Number of cells with valid core** | 137,024 | 113,392 | 283,359 | 533,775 |
| **Amount of analyzed area (mm^2^)** | 37.08 | 25.92 | 57.96 | 120.96 |
| **Amount of analyzed area  with valid core (mm^2^)** | 27.72 | 23.76 | 57.96 | 109.44 |
