# Supplementary material for: Spatial Landscape of Malignant Pleural and Peritoneal Mesothelioma Tumor Immune Microenvironments
Source: Cancer Res Commun. 2024 Aug 16;4(8):2133–46. doi: 10.1158/2767-9764.CRC-23-0524 (PMC11328914; doi:10.1158/2767-9764.CRC-23-0524)
Supplement: Supplementary Table 3 — Comparison of differences in cell–cell contact scores between malignant pleural mesothelioma (MPM) and malignant peritoneal mesothelioma (MPeM) tumors. [file crc-23-0524_supplementary_table_3_suppst3.docx]

**Supplementary Table 3: Comparison of differences in cell–cell contact scores between malignant pleural mesothelioma (MPM) and malignant peritoneal mesothelioma (MPeM) tumors.**

| **Contact pair** | ***P*-value** | **FDR-adjusted** | **Mean contact score difference (MPM – MPeM)** |
| --- | --- | --- | --- |
| Tumor cells – tumor cells | 0.0002 | 0.0036 | 0.091 |
| CD8^+^ T cells – CD8^+^ T cells | 0.0012 | 0.0125 | -0.095 |
| CD4^+^ T cells – tumor cells | 0.0112 | 0.0784 | 0.058 |
| Macrophages – tumor cells | 0.0158 | 0.0830 | 0.035 |
| Tregs – tumor cells | 0.0300 | 0.1054 | 0.041 |
| CD8^+^ T cells – tumor cells | 0.0301 | 0.1054 | 0.043 |
| B cells – Macrophages | 0.0393 | 0.1118 | -0.214 |
| B cells – CD8^+^ T cells | 0.0426 | 0.1118 | -0.051 |
| CD8^+^ T cells – Macrophages | 0.0625 | 0.1458 | -0.069 |
| Macrophages – Macrophages | 0.0714 | 0.1499 | 0.026 |
| CD8^+^ T cells – FOXP3^+^ | 0.1020 | 0.1947 | -0.042 |
| CD4^+^ T cells – CD8^+^ T cells | 0.1180 | 0.2065 | -0.040 |
| B cells – B cells | 0.1620 | 0.2460 | -0.026 |
| B cells – CD4^+^ T cells | 0.1640 | 0.2460 | -0.094 |
| B cells – tumor cells | 0.2280 | 0.3192 | 0.072 |
| Tregs – Tregs | 0.4020 | 0.5276 | 0.045 |
| CD4^+^ T cells – Macrophages | 0.4550 | 0.5621 | -0.014 |
| CD4^+^ T cells – Tregs | 0.5730 | 0.6540 | 0.014 |
| CD4^+^ T cells – CD4^+^ T cells | 0.5930 | 0.6540 | 0.022 |
| Tregs – Macrophages | 0.6240 | 0.6540 | 0.009 |
| B cells – Tregs | 0.6540 | 0.6540 | 0.097 |

Statistical analysis was conducted using two-sided Kruskal–Wallis tests, and significance was determined with FDR-adjusted *P*-values <0.15.
