# Supplementary material for: Spatial Landscape of Malignant Pleural and Peritoneal Mesothelioma Tumor Immune Microenvironments
Source: Cancer Res Commun. 2024 Aug 16;4(8):2133–46. doi: 10.1158/2767-9764.CRC-23-0524 (PMC11328914; doi:10.1158/2767-9764.CRC-23-0524)
Supplement: Supplementary Table 4 — Distribution of immune cells and tumor cells across different histologies in the malignant mesothelioma (MM) cohort. [file crc-23-0524_supplementary_table_4_suppst4.docx]

**Supplementary Table 4: Distribution of immune cells and tumor cells across different histologies in the malignant mesothelioma (MM) cohort.**

| **Cell Type** | **MPM** | | | **MPeM** |
| --- | --- | --- | --- | --- |
|  | **Epithelioid vs. Biphasic** | **Epithelioid vs Sarcomatoid** | **Biphasic vs Sarcomatoid** | **Epithelioid vs. Biphasic** |
| B cells (CD20^+^) | **0.044** | 0.095 | 0.416 | 0.217 |
| CD4^+^ T cells | 0.371 | **0.037** | **0.040** | 0.473 |
| CD8^+^ T cells | 0.053 | 0.475 | 0.077 | 0.420 |
| Tregs (FOXP3^+^ CD4^+^) | 0.163 | 0.256 | 0.447 | 0.469 |
| Macrophages (CD68^+^) | 0.366 | 0.282 | 0.337 | 0.101 |
| DCs (CD11c^+^) | 0.410 | 0.677 | 0.637 | 0.230 |
| NK (CD56^+^) | 0.161 | 0.323 | 0.108 | 0.319 |
| Pan-CK^+^ | 0.491 | **0.001** | **0.001** | 0.420 |

Data represent P-values for individual immune and tumor cell subsets. Statistical analysis was performed using two-sided Kruskal-Wallis tests. *P*<0.05 was considered significant. Malignant pleural mesothelioma (MPM) and malignant peritoneal mesothelioma (MPeM).
