# Supplementary material for: Spatial Landscape of Malignant Pleural and Peritoneal Mesothelioma Tumor Immune Microenvironments
Source: Cancer Res Commun. 2024 Aug 16;4(8):2133–46. doi: 10.1158/2767-9764.CRC-23-0524 (PMC11328914; doi:10.1158/2767-9764.CRC-23-0524)
Supplement: Supplementary Table 5 — Comparison of cell type distributions between malignant pleural mesothelioma (MPM) and malignant peritoneal mesothelioma (MPeM) within the same histologic subtypes. [file crc-23-0524_supplementary_table_5_suppst5.docx]

**Supplementary Table 5: Comparison of cell type distributions between malignant pleural mesothelioma (MPM) and malignant peritoneal mesothelioma (MPeM) within the same histologic subtypes.**

| **Cell Type** | **MPM vs. MPeM** | |
| --- | --- | --- |
|  | **Epithelioid** | **Biphasic** |
| B cells (CD20^+^) | 0.234 | 0.122 |
| CD4^+^ T cells | 0.531 | 0.184 |
| CD8^+^ T cells | 0.065 | 0.479 |
| Tregs (FOXP3^+^ CD4^+^) | 0.206 | 0.077 |
| Macrophages (CD68^+^) | 0.625 | 0.093 |
| DCs (CD11c^+^) | 0.689 | 0.246 |
| NK (CD56^+^) | 0.342 | 0.521 |
| Pan-CK^+^ | 0.072 | 0.184 |

Data are presented as *P*-values of differences for individual immune cell and tumor cell populations between MPM and MPeM tumors. Statistical analysis was performed using one-sided Kruskal-Wallis tests. *P*<0.05 was considered significant.
