# Supplementary material for: Spatial Landscape of Malignant Pleural and Peritoneal Mesothelioma Tumor Immune Microenvironments
Source: Cancer Res Commun. 2024 Aug 16;4(8):2133–46. doi: 10.1158/2767-9764.CRC-23-0524 (PMC11328914; doi:10.1158/2767-9764.CRC-23-0524)
Supplement: Supplementary Table 6 — The association of immune cells and cellular neighborhood proportion with overall survival in all malignant mesothelioma (MM) as well as for malignant pleural mesothelioma (MPM) and malignant peritoneal mesothelioma (MPeM) separately. [file crc-23-0524_supplementary_table_6_suppst6.docx]

**Supplementary Table 6: The association of immune cells and cellular neighborhood proportion with overall survival in all malignant mesothelioma (MM) as well as for malignant pleural mesothelioma (MPM) and malignant peritoneal mesothelioma (MPeM) separately.**

| **Cell type** | **MM** | | **MPM** | | **MPeM** | |
| --- | --- | --- | --- | --- | --- | --- |
|  | **hazard** | **P value** | **hazard** | **P value** | **hazard** | **P value** |
| CD4+ T cells | 1.04E-01 | 1.70E-01 | 3.29E+00 | 5.04E-01 | 5.38E-06 | **1.43E-02** |
| CD8+  T cells | 7.05E-01 | 6.43E-01 | 5.80E-01 | 4.81E-01 | 2.02E-01 | 5.63E-01 |
| Pan-CK+ | 5.12E-01 | 2.70E-01 | 4.07E-01 | 1.68E-01 | 2.93E+00 | 4.89E-01 |
| B cells  (CD20+) | 4.61E-03 | 4.57E-01 | 7.98E-04 | 3.29E-01 | 6.06E-07 | 6.89E-01 |
| Tregs  (FOXP3+ CD4+) | 4.33E-03 | 2.60E-01 | 6.19E-04 | 1.37E-01 | 1.06E+02 | 7.68E-01 |
| Macrophages  (CD68+) | 8.34E-01 | 8.28E-01 | 1.55E+00 | 7.26E-01 | 1.07E+00 | 9.55E-01 |
| DCs  (CD11c^+^) | 3.67E+00 | 4.20E-01 | 2.37E+00 | 6.08E-01 | 1.97E+00 | 8.97E-01 |
| NK  (CD56^+^) | 3.33E+00 | 5.88E-01 | 2.73E+00 | 6.75E-01 | 9.40E-01 | 9.91E-01 |
| CN1 | 7.49E-01 | 6.62E-01 | 2.64E-01 | 9.95E-02 | 1.42E+01 | 1.08E-01 |
| CN2 | 1.32E+00 | 4.06E-01 | 1.14E+00 | 7.10E-01 | 2.69E-01 | 5.59E-01 |
| CN3 | 1.59E+00 | 3.28E-01 | 2.99E+00 | **1.47E-02** | 3.45E-03 | **3.00E-02** |
| CN4 | 1.27E+00 | 7.40E-01 | 2.31E+00 | 2.94E-01 | 8.02E-01 | 8.84E-01 |
| CN5 | 6.11E-01 | 1.60E-01 | 4.80E-01 | 7.00E-02 | 1.79E+00 | 4.68E-01 |
| CN6 | 1.09E+00 | 9.04E-01 | 1.70E+00 | 5.88E-01 | 1.62E+00 | 7.10E-01 |

Data are presented as *P*-values based on log-rank tests. *P*<0.05 was considered significant.
