# Supplementary material for: Spatial Landscape of Malignant Pleural and Peritoneal Mesothelioma Tumor Immune Microenvironments
Source: Cancer Res Commun. 2024 Aug 16;4(8):2133–46. doi: 10.1158/2767-9764.CRC-23-0524 (PMC11328914; doi:10.1158/2767-9764.CRC-23-0524)
Supplement: Supplementary Table 7 — Comparison of clinical parameters between malignant pleural mesothelioma (MPM) and malignant peritoneal mesothelioma (MPeM) for various cell types. [file crc-23-0524_supplementary_table_7_suppst7.docx]

**Supplementary Table 7: Comparison of clinical parameters between malignant pleural mesothelioma (MPM) and malignant peritoneal mesothelioma (MPeM) for various cell types.**

|  | **CD4^+^**  **T cells** | **CD8^+^**  **T cells** | **Pan-CK^+^** | **B cells**  **(CD20^+^)** | **Tregs**  **(FOXP3^+^ CD4^+^)** | **Macrophages (CD68^+^)** | **DCs (CD11c^+^)** | **NK**  **(CD56^+^)** |
| --- | --- | --- | --- | --- | --- | --- | --- | --- |
| High-grade | 0.680 | 0.061 | 0.054 | 0.897 | 0.696 | 0.827 | 0.266 | 0.725 |
| Low/Median-grade | NA | NA | NA | NA | NA | NA | NA | NA |
| Smoker | 0.876 | **0.131** | **0.071** | 0.897 | 0.696 | 0.592 | 0.515 | 0.722 |
| Non-smoker | 0.876 | **0.131** | 0.204 | 0.897 | 0.696 | 0.827 | 0.847 | 0.725 |
| Asbestos Exposure | **0.065** | **0.123** | **0.136** | 0.897 | 0.696 | 0.827 | 0.818 | 0.722 |
| No Asbestos Exposure | NA | NA | NA | NA | NA | NA | NA | NA |
| Young  (< 60 years of age) | 0.876 | **0.091** | **0.122** | 0.897 | 0.721 | 0.592 | 0.815 | 0.725 |
| Elderly (>60 years of age) | 0.876 | **0.002** | **0.042** | 0.897 | 0.696 | 0.495 | **0.065** | **0.153** |
| White | 0.876 | **0.002** | **0.023** | 0.897 | 0.696 | 0.495 | **0.169** | 0.725 |
| Non-White | NA | NA | NA | NA | NA | NA | NA | NA |
| Male | 0.680 | **0.011** | **0.071** | 0.897 | 0.696 | 0.964 | 0.818 | 0.510 |
| Female | 0.680 | **0.131** | **0.023** | 0.897 | 0.696 | 0.592 | 0.266 | 0.153 |

FDR-corrected two-sided Wilcox test for sex, age, smoking status, asbestos exposure, and stage. Values with FDR <0.15 are in bold. NA: Not applicable.
