# Supplementary material for: Spatial Landscape of Malignant Pleural and Peritoneal Mesothelioma Tumor Immune Microenvironments
Source: Cancer Res Commun. 2024 Aug 16;4(8):2133–46. doi: 10.1158/2767-9764.CRC-23-0524 (PMC11328914; doi:10.1158/2767-9764.CRC-23-0524)
Supplement: Supplementary Table 8 — Comparison of cell–cell contact score differences between malignant pleural mesothelioma (MPM) and malignant peritoneal mesothelioma (MPeM). [file crc-23-0524_supplementary_table_8_suppst8.docx]

**Supplementary Table 8: Comparison of cell–cell contact score differences between malignant pleural mesothelioma (MPM) and malignant peritoneal mesothelioma (MPeM).**

|  | **MPM** | | | **MPeM** | | |
| --- | --- | --- | --- | --- | --- | --- |
| **Contact pair** | ***P*-value** | **FDR-adjusted** | **BAP1-high – BAP1-low** | ***P*-value** | **FDR-adjusted** | **BAP1-high – BAP1-low** |
| CD4^+^ T cells – tumor cells | 0.074 | 0.120 | -0.685 | 0.549 | 0.549 | 0.226 |
| CD8^+^ T cells – tumor cells | **0.003** | **0.013** | -0.521 | **0.046** | **0.093** | -0.630 |
| Macrophages – tumor cells | 0.090 | 0.120 | -0.147 | 0.036 | 0.093 | 0.756 |
| B cells – tumor cells | NA | NA | -0.147 | NA | NA | 0.756 |
| Tregs – tumor cells | 0.500 | 0.500 | -0.460 | 0.500 | 0.549 | -0.199 |

Statistical analysis was performed using two-sided Kruskal-Wallis tests with FDR adjustments. *P*<0.15 was considered significant.
